# The Relationship between Gestational Weight Gain and Postpartum Depression in Normal and Overweight Pregnant Women

**DOI:** 10.1155/2018/9315320

**Published:** 2018-10-21

**Authors:** Fatemeh Dayan, Nahid Javadifar, Mitra Tadayon, Amal Saki Malehi, Hosein Komeili Sani

**Affiliations:** ^1^Department of Midwifery, Ahvaz Jundishapur University of Medical Sciences, Ahvaz, Iran; ^2^Reproductive Health Promotion Research Centre, Ahvaz Jundishapur University of Medical Sciences, Ahvaz, Iran; ^3^Department of Biostatistics and Epidemiology, Ahvaz Jundishapur University of Medical Sciences, Ahvaz, Iran; ^4^Department of Nursing, Ahvaz Jundishapur University of Medical Sciences, Ahvaz, Iran

## Abstract

**Objective:**

To investigate the relationship between weight gain in pregnancy and postpartum depression (PPD) in normal and overweight pregnant women.

**Methods:**

The participants of this prospective cohort study were 223 healthy pregnant women with the first trimester body mass index (BMI) between 18.5 and 30 and the gestational age of 10-14 weeks and depressed women were excluded with Beck questionnaire in the first trimester. The evaluation included weight gain at the end of the second and third trimesters and the screening of PPD in 6-8 weeks after delivery by Edinburgh scale.

**Results:**

49 participants were excluded from the study and data from 174 people were analyzed. 32.2% of mothers were scored above 12 in the Edinburgh scale. The only variable associated with depression was the third trimester weight gain (OR 1.17, 95%CI 1.04-1.32).

**Conclusion:**

In addition to considering other risk factors for postpartum depression, health care providers should consider the higher probability of PPD in prepregnancy normal and overweight women who have excessive weight gain especially in the third trimester of pregnancy.

## 1. Introduction

Pregnancy and postpartum period are known as times when the mood vulnerability is created [1]. These disorders have different patterns and can disrupt the self-care process of women and have a negative effect on their maternal role after delivery. One of the most common mood disorders in women after childbirth is postpartum depression (PPD). Despite some disagreements about the postpartum depression period, diagnostic and statistical manual of mental disorders has defined it as a major depressive event occurring in the first 4 weeks after childbirth [2].

There are various estimates for the prevalence of PPD in the world. For example, this rate is 14.6% in the United States [3], 34% in Jamaica [4], and 28% in Pakistan [5]. A systematic review in Iran showed that the prevalence of this disorder was 28.7%, which indicates its relatively high prevalence in Iran [6].

One of the factors affecting depression is obesity [7–9]. Studies have shown that, in obese people, fatty tissue secrets inflammatory hormone. This is similar to a chronic inflammatory condition that can be associated with depression [10]. Also, concerns about beauty and apparent changes can produce anxiety and depression especially in pregnant women due to weight gain [11]. The average weight gain at pregnancy is 12 kg. The weight gain for pregnant women is estimated based on body mass index (BMI). Using this index at the beginning of pregnancy, the weight of the mother is categorized into four groups: underweight, normal, overweight, and obese. About 46% of women have weight changes outside the recommended range [12].

Although few studies have been conducted in developing countries [1], most studies have examined the relationship between obesity in pregnancy and PPD. There is a dearth study on weight gain during pregnancy and PPD [13] and there are few studies that focus on the role of weight gain in those who were not obese or depressed before pregnancy. Despite the prevalence of PPD and its long-term effects on the family, the disorder has been repeatedly neglected by patients and their health care providers, and only a small number of them are identified and treated. Since the identification of factors affecting PPD, one of which may be weight gain during pregnancy, is helpful in management of this disease, the aim of this study has been to investigate the association between postpartum depression and weight gain of normal and overweight pregnant women.

## 2. Material and Methods

This is a prospective cohort study approved by Ethics Committee of Ahwaz University of Medical Sciences. During a 3-month period from October to December 2015, 223 pregnant women who were eligible and willing to participate in the study attended to the selected health care centers of Ahwaz. The city of Ahvaz has two eastern and western parts. Six health care centers were selected randomly from both eastern (3 centers) and western parts (3 centers). The sample size for the present study was determined based on a relevant study with odds ratio of 2 [14] and considering a power of 80%, significance level of 5%, and the attrition rate of 30% (n=110) and then the sample size was doubled to ensure a conservative sample size that could be achieved during the study [15]. In this study, all pregnant mothers who met the inclusion criteria and attended to these centers within 3 months (October to December 2016) were selected by convenient sampling method. The inclusion criteria were healthy pregnant women aged 18-35 years, gravid of 1-5, BMI between 18.5 and 30, and gestational age of 10-14 weeks based on LMP or ultrasound of early pregnancy. Exclusion criteria were preterm delivery, underlying disease during pregnancy, stillbirth, and divorce as well as the occurrence of severe stressful situations in life, such as death of close relatives, domestic violence, and positive results from Beck depression test at the beginning of the study. Studies have shown that any history of depression is one of the biggest risk factors for postpartum depression [16, 17]. The Beck depression inventory contains 21 symptoms and a score above 11 was considered as depression. The validity of this questionnaire was confirmed in Iran by Rajabi et al. with Cronbach's Alpha of 87% [18].

After obtaining written consent and negative results from the depression test and completion of the demographic questionnaire, the weight was measured at the end of the second (weeks 26-28) and the third trimesters (weeks 38-40). In order to calculate BMI (kg/m^2^) of mothers in the first evaluation at 10-14 weeks, the height and weight were measured by fabric yardstick with standard deviation of 0.5 cm and light clothing without shoes by using valid and identical scales in each center with a standard deviation of 0.1 kg, that were calibrated at regular intervals by a standard weigh. The pattern of gestational weight gain (GWG) is related to prepregnancy BMI or maternal BMI calculated in the first visit in the first trimester of pregnancy. Based on the recommendations of the Institute of medicine (IOM), the weight gain recommended for normal weight pregnant mothers (BMI = 18.5-24.9) and overweight pregnant women (BMI = 25-29.9) are 11.5- 16 kg and 7-11.5 kg, respectively. According to the IOM, inadequate and above optimal weight gains are respectively less than 11.5 and above 16 kg for mothers with normal weight, less than 7 and more than 11.5 Kg for overweight mothers [19]. Advice and training on proper weight gain and nutrition during pregnancy were routinely provided to all pregnant mothers. These mothers were also examined after delivery in the days 45-60 using the Persian version of the Edinburgh scale whose validity was confirmed in Iran by Khyberabadi et al. (2012) with Cronbach's alpha of 0.79 and sensitivity of 78% and specificity of 75% respectively. The Edinburgh scale is a self-report questionnaire containing 10 items with 4 options, the result of which is between 0 and 30 points, and the score above 12 is the cut off for diagnosing PPD [20]. All mothers who received positive results of Beck test at the beginning of pregnancy as well as mothers with positive results from Edinburgh postpartum depression test were referred to mental health services in health care centers.

In this study, quantitative variables are presented in terms of mean and standard deviation and stratified variables in terms of numbers and percentages. T-test was used to compare the means and logistic regression analysis was used to evaluate the independent variables in postpartum depression. The significance level was considered as 0.05.

## 3. Results

After collecting data, results obtained from 174 pregnant women entered statistical analysis. 23 pregnant women were excluded from the study due to positive results from the Beck test. 9 participants with gestational diabetes mellitus, 2 cases of hypothyroidism, and 5 patients with preterm labor were hospitalized. 9 people did not attend follow-up and did not respond to telephone calls, 1 had a stillbirth, and thus 49 participants were excluded from the study. The mean age of the participants was 25.7 years. 42% of the mothers experienced the first pregnancy. 67.8% of the mothers were scored below 12 in Edinburgh's postpartum depression scale and 32.2% had a score above 12. After examining the demographic variables between the two depressed and nondepressed groups including mother's age, education level, job, type of delivery, sex of neonate, number of pregnancies, and father's education and job, the household income was the only variable that had significant difference in the two groups and it was lower in the depressed mothers (p <0.05) (Table 1).

The data related to the main variables including prepregnancy weights, body mass index, the second and third trimester weight gain, and type and total weight gain in pregnancy were also statistically analyzed. To measure them, the statistical test of logistic regression was used and there was a significant relationship between depression and weight gain of the 3rd trimester (OR = 1.17).

According to the amounts recommended by the IOM for normal and overweight pregnant women, weight changes were divided into 3 groups: inadequate, adequate, and excessive, and its relationship with depression was investigated by logistic regression test which did not show any significant relationship (Table 2). Since income was significantly different between depressed and nondepressed groups, the relationship between depression and all weight variables was remeasured with modified effect of income and again only the third trimester weight gain had significant relationship with depression considering the effect of income (OR 1.17, 95%CI 1.04-1.32) (Table 3).

## 4. Discussion

As shown in the present study, there was no relationship between initial weight and initial BMI as well as the type of BMI (normal or overweight) and PPD. Evidence revealed that women in gestational period are at the higher risk of depression and studies have shown a relationship between obesity and depression in pregnant and nonpregnant women and concluded that there is a dose-response relationship between the increase in primary BMI and the increased risk of depression during pregnancy and postpartum [1].

In the present study, 33% of mothers with normal BMI and 31.4% of overweight pregnant women had positive results of PPD screening, although the two groups did not have a significant difference in postpartum depression.

Consistent with our findings, LaCoursiere (2006) showed that the prevalence of PPD symptoms in women with normal BMI was 22.8 ± 1.2% and it was 30.8%  ± 2.5% in overweight women [14] but in an another study, among 245 pregnant women with normal weight during pregnancy, 7.8% in the 6-8 weeks after delivery had depression [21]. We hypothesized that the more BMI at the beginning of pregnancy is, the more probably the mother would be to experience PPD but the data did not support the hypothesis. Fox and Yamaguchi (1997) concluded that in comparison with prepregnancy normal weight women, overweight women were more likely to have positive view about their body image and weight gain during pregnancy [22] and the rates of experience PPD are similar for the two groups [13].

In the present study, neither weight gain in the second trimester nor the mean of GWG during pregnancy associated with PPD. The only relationship was between the weight gain in the third trimester and the increased risk of PPD (OR 1.17, 95%CI 1.04-1.32), so that the increase in each kilogram in the third trimester weight gain increased the chance of depression by 0.17.

However, in many studies, there is a positive correlation between GWG in the second and third trimesters and pregnancy outcomes such as birth weight and duration of pregnancy, but few studies have considered weight gain in each trimester [23, 24]. We did not find a study comparing weight gain in the second and third trimesters as well as the mean weight gain based on weight and BMI at the beginning of pregnancy in terms of the presence or absence of PPD. There was no significant difference in pattern of weight gain in this study. Overall, 52.9% of participants had good weight gain and 15.5% were higher than recommended weights. About 46% of women have weight changes outside the recommended range [12]. In the study by Dimert et al. (2016), 40% of mothers had good weight gain and 22% had more than recommended weights [25]. The weight gain pattern is depending on the prepregnancy BMI, and can vary by race and age of mother [23]. Another study results suggest that there is no relationship between BMI and weight gain in predicting the symptoms of depression in pregnancy [26].

This study showed that about one-third of mothers who were nondepressed at the beginning of pregnancy and were normal or overweight had PPD according to Edinburgh test results. Based on evidence, a PPD can be associated with prepregnancy depression [27, 28], and in the present study, the participants did not have any positive history of depression and they obtained negative results of Beck depression screening in the first trimester. In spite of limiting initial pregnancy BMI in to the two groups, this result is in accordance with total prevalence of PPD in previous studies in Iran [6, 29].

Comparison of demographic variables in two groups of depressed and nondepressed mothers showed that among the confounding factors, only household income was significantly different in both groups and was lower in depressed mothers. Some studies have shown that there is a relationship between postpartum depression and income or other demographic factors, and in some cases, such a relationship has not been found which could be influenced by many factors, including the type and conditions of the study [27, 30, 31].

Considering the existence of sufficient evidence for the relationship between early obesity or underweight in pregnancy and postpartum depression, this study showed significant evidence for the association between the amount of BMI at the beginning of pregnancy and the weight gain in the whole pregnancy and its trimesters in normal and overweight women. One of advantages of this study is the longitudinal conduction of the study and the exclusion of depressed women at the beginning of pregnancy that has been considered as a research limitation in many studies. However, the limitations of this study can also be the lack of evaluation of important factors such as the level of perceived social support and the support of the spouse, as well as the degree of adaptation to maternal role that could be contributed to the occurrence of postpartum depression.

## 5. Conclusion

Based on the results of this study, it seems that one of the predictive variables of postpartum depression in normal and overweight pregnant women is the weight gain in the third trimester of pregnancy. Therefore, health care providers, in addition to considering other risk factors for postpartum depression, should consider the higher probability of PPD in prepregnancy normal and overweight women who have excessive weight gain especially in the third trimester of pregnancy.

## Figures and Tables

**Table 1:** Characteristics of participants in depressed and nondepressed groups.

Variable		Total n (%)	Non- depressed (n%)	Depressed (n%)	P- value
Education	<6	51 (29.3)	39 (22.41)	12 (6.89)	0.098
(year)	6-9	33 (19)	17 (9.77)	16 (9.19)	
	9-12	63 (36.2)	42 (24.1)	21 (12.06)	
	university	27 (15.5)	20 (11.49)	7 (4.02)	

Type of delivery	NVD	119 (68.4)	79 (45.4)	40 (22.98)	0.553
	C/S	55 (31.6)	39 (22.41)	16 (9.19)	

Sex of neonate	Female	90 (51.7)	58 (33.33)	32 (18.39)	0.324
	Male	84 (48.3)	60 (33.48)	24 (13.79)	

Gravid	1	73 (42)	48 (27.58)	25 (4.36)	0.956
	2	59 (33.9)	40 (22.98)	19 (10.91)	
	3	29 (16.7)	20 (11.49)	9 (5.17)	
	4	9 (5.2)	7 (4.02)	2 (1.14)	
	5	4 (2.3)	3 (1.72)	1 (.57)	

Employment status	House wife	158 (90.8)	110 (63.21)	48 (27.58)	0.158
	Employed	16 (9.2)	8 (4.59)	8 (4.59)	

Education of spouse	<6	27 (15.5)	19 (10.91)	8 (4.59)	0.170
(year)	6-9	48 (27.6)	30 (17.24)	18 (10.34)	
	9-12	68 (39.1)	43 (24.71)	25 (14.36)	
	University	31 (17.8)	26 (14.94)	5 (2.87)	

Employment of spouse	Unemployed	16 (9.2)	11 (6.32)	5 (2.87)	0.84
	Worker	39 (22.4)	27 (15.51)	12 (6.89)	
	Employee	17 (9.8)	13 (7.47)	4 (2.29)	
	Free job	102 (58.6)	67 (38.55)	35 (20.11)	

Age (mean) (18-35 yr)	-	25.7 ± 3.1	25.2 ± 2.1	26.6 ± 2.7	0.57

Family income (tuman^1^) (mean)	-	1320000 ± 97000	1640000 ± 112000	1014000 ± 83000	0.046

*Note. *Values are expressed as means and standard deviations or number and percentage.

1 At the time of data collection, US$1 = 3,400 Tuman.

**Table 2:** Odds ratios for type of weight gain and BMI by depression.

Variable		Non depressed n (%)	Depressed n (%)	OR	CI	p-value
Type of weight gain (kg)	Inadequate	42 (24%)	13 (7.4%)	0.55	0.24-1.60	0.326
	Adequate	59 (33.9%)	33 (19%)	1.05	0.21-1.57	0.281
	Excessive	17 (9.7%)	10 (5.7%)	Ref.		

Type of BMI (kg/m^2^)	Normal	59 (33.9%)	29 (16.6%)	0.82	0.56-2.03	0.826
	Over weight	59 (33.9%)	27 (15.5%)	Ref.		

**Table 3:** Adjusted odds ratio of pregnancy weight gain and PPD.

Variable	Coefficient of regression	OR	CI	P-value
Initial weight (kg)	-0.004	0.99	0.96-1.02	0.563
Body mass index (kg/m^2^)	-0.2	0.97	0.87-1.07	0.545
Second trimester weight gain (kg)	-0.26	0.974	0.87-1/08	0.629
Third trimester weight gain (kg)	0.163	1.17	1.04-1.32	0.007
Total weight gain (kg)	0.080	1.08	0.98-1.18	0.084
